# Supplementary material for: Beta turn propensity and a model polymer scaling exponent identify intrinsically disordered phase-separating proteins
Source: J Biol Chem. 2021 Oct 26;297(5):101343. doi: 10.1016/j.jbc.2021.101343 (PMC8592878; doi:10.1016/j.jbc.2021.101343)
Supplement: Figures S1–S7 and Tables S1–S5 [file mmc1.docx]

**Supporting Information for:**

**Beta turn propensity and a model polymer scaling exponent identify intrinsically disordered phase-separating proteins**

*Elisia A. Paiz, Jeffre H. Allen, John J. Correia, Nicholas C. Fitzkee, Loren E. Hough, and Steven T. Whitten*

**Contents:**

Supporting Tables

S1. List of IDPs with experimental mean *R_h_*.

S2. List of proteins that exhibit phase separation behavior.

S3. Summary of Mann-Whitney U tests that compare mean *v_model_* (top) and mean β-turn propensity (bottom) in the null, testing, and folded sets.

S4. Normalized frequency for β-turn.

S5. Structural properties of turn and non-turn ensembles.

S6. List of proteins that exhibit phase separation behavior *in cellulo* that were found by *in vitro* characterization not to phase separate as purified proteins.

S7. Summary of pair-wise Mann-Whitney U tests comparing the relative population (given by set percentage) of predicted PS region lengths, for lengths ranging from 1 to 150 residues.

Supporting Figures

S1. Experimental mean *R_h_* compared to sequence calculated mean *R_h_*.

S2. Distribution of *v_model_* values in the A) testing and B) null sets.

S3. Comparing sequence calculated *v_model_* and *v_int_*.

S4. Predicting protein regions that drive LLPS.

S5. Pair-wise correlations of predictor results.

Supporting References

**Supporting Tables**

**Table S1. List of IDPs with experimental mean *R_h_*.**

| **Name** | ***N*** | **mean *R_h_* (Å)** | **sequence** | **reference** |
| --- | --- | --- | --- | --- |
| p53(1-93) | 95 | 32.4 | GSMEEPQSDPSVEPPLSQETFSDLWKLLPENNVLSPLPSQAMDDLMLSPDDIEQWFTEDPGPDEAPRMPEAAPPVAPAPAAPTPAAPAPAPSWPL | (1) |
| p53(1-73) TAD | 73 | 23.8 | MEEPQSDPSVEPPLSQETFSDLWKLLPENNVLSPLPSQAMDDLMLSPDDIEQWFTEDPGPDEAPRMPEAAPRV | (2) |
| Vmw65 | 89 | 28 | GSAGHTRRLSTAPPTDVSLGDELHLDGEDVAMAHADALDDFDLDMLGDGDSPGPGFTPHDSAPYGALDMADFEFEQMFTDALGIDEYGG | (3) |
| Hdm2-ABD | 97 | 31.7 | ERSSSSESTGTPSNPDLDAGVSEHSGDWLDQDSVSDQFSVEFEVESLDSEDYSLSEEGQELSDEDDEVYQVTVYQAGESDTDSFEEDPEISLADYWK | (4) |
| prothymosin-α | 110 | 33.6 | MSDAAVDTSSEITTKDLKEKKEVVEEAENGRDAPANGNANEENGEQEADNEVDEEEEEGGEEEEEEEEGDGEEEDGDEDEEAESATGKRAAEDDEDDDVDTKKQKTDEDD | (4) |
| HIF1-α-403 | 202 | 44.3 | PAAGDTIISLDFGSNDTETDDQQLEEVPLYNDVMLPSPNEKLQNINLAMSPLPTAETPKPLRSSADPALNQEVALKLEPNPESLELSFTMPQIQDQTPSPSDGSTRQSSPEPNSPSEYCFYVDSDMVNEFKLELVEKLFAEDTEAKNPFSTQDTDLDLEMLAPYIPMDDDFQLRSFDQLSPLESSSASPESASPQSTVTVFQ | (5) |
| Fos-AD | 168 | 35 | GSHMSVASLDLTGGLPEVATPESEEAFTLPLLNDPEPKPSVEPVKSISSMELKTEPFDDFLFPASSRPSGSETARSVPDMDLSGSFYAADWEPLHSGSLGMGPMATELEPLCTPVVTCTPSCTAYTSSFVFTYPEADSFPSCAAAHRKGSSSNEPSSDSLSSPTLLAL | (6) |
| Mlph(147-240) | 97 | 28 | RLQGGGGSEPSLEEGNGDSEQTDEDGDLDTEARDQPLNSKKKKRLLSFRDVDFEEDSDHLVQPCSQTLGLSSVPESAHSLQSLSGEPYSEDTTSLEP | (7) |
| tau-K45 | 198 | 45 | MSSPGSPGTPGSRSRTPSLPTPPTREPKKVAVVRTPPKSPSSAKSRLQTAPVPMPDLKNVKSKIGSTENLKHQPGGGKVQIINKKLDLSNVQSKCGSKDNIKHVPGGGSVQIVYKPVDLSKVTSKCGSLGNIHHKPGGGQVEVKSEKLDFKDRVQSKIGSLDNITHVPGGGNKKIETHKLTFRENAKAKTDHGAEIVY | (8) |
| Mlph(147-403) | 260 | 49 | RLQGGGGSEPSLEEGNGDSEQTDEDGDLDTEARDQPLNSKKKKRLLSFRDVDFEEDSDHLVQPCSQTLGLSSVPESAHSLQSLSGEPYSEDTTSLEPEGLEETGARALGCRPSPEVQPCSPLPSGEDAHAELDSPAASCKSAFGTTAMPGTDDVRGKHLPSQYLADVDTSDEDSIQGPRAASQHSKRRARTVPETQILELNKRMSAVEHLLVHLENTVLPPSAQEPTVETHPSADTEEETLRRRLEELTSNISGSSTSSE | (7) |
| p57-ID | 73 | 24 | VRTSACRSLFGPVDHEELSRELQARLAELNAEDQNRWDYDFQQDMPLRGPGRLQWTEVDSDSVPAFYRETVQV | (9) |
| PDE-γ | 87 | 24.8 | MNLEPPKAEIRSATRVMGGPVTPRKGPPKFKQRQTRQFKSKPPKKGVQGFGDDIPGMEGLGTDITVICPWEAFNHLELHELAQYGII | (10) |
| LJIDP1 | 94 | 24.52 | MARSFTNIKAISALVAEEFSNSLARRGYAATAQSAGRVGASMSGKMGSTKSGEEKAAAREKVSWVPDPVTGYYKPENIKEIDVAELRSAVLGKN | (11) |
| cad136 | 136 | 28.1 | RLEQYTSAVVGNKAAKPAKPAASDLPVPAEGVRNIKSMWEKGNVFSSPGGTGTPNKETAGLKVGVSSRINEWLTKTPEGNKSPAPKPSDLRPGDVSGKRNLWEKQSVEKPAASSSKVTATGKKSETNGLRQFEKEP | (12) |
| α-synuclein | 140 | 28.2 | MDVFMKGLSKAKEGVVAAAEKTKQGVAEAAGKTKEGVLYVGSKTKEGVVHGVATVAEKTKEQVTNVGGAVVTGVTAVAQKTVEGAGSIAAATGFVKKDQLGKNEEGAPQEGILEDMPVDPDNEAYEMPSEEGYQDYEPEA | (13) |
| CFTR-R-region | 189 | 32 | GAMESAERRNSILTETLHRFSLEGDAPVSWTETKKQSFKQTGEFGEKRKNSILNPINSIRKFSIVQKTPLQMNGIEEDSDEPLERRLSLVPDSEQGEAILPRISVISTGPTLQARRRQSVLNLMTHSVNQGQNIHRKTTASTRKVSLAPQANLTELDIYSRRLSQETGLEISEEINEEDLKECLFDDME | (14) |
| SNAP25 | 206 | 39.7 | MAEDADMRNELEEMQRRADQLADESLESTRRMLQLVEESKDAGIRTLVMLDEQGEQLERIEEGMDQINKDMKEAEKNLTDLGKFCGLCVCPCNKLKSSDAYKKAWGNNQDGVVASQPARVVDEREQMAISGGFIRRVTNDARENEMDENLEQVSGIIGNLRHMALDMGNEIDTQNRQIDRIMEKADSNKTRIDEANQRATKMLGSG | (15) |
| ShB-C | 146 | 32.9 | MTLGQHMKKSSLSESSSDMMDLDDGVESTPGLTETHPGRSAVAPFLGAQQQQQQPVASSLSMSIDKQLQHPLQQLTQTQLYQQQQQQQQQQQNGFKQQQQQTQQQLQQQQSHTINASAAAATSGSGSSGLTMRHNNALAVSIETDV | (16) |
| HIF1-α-530 | 170 | 38.3 | NEFKLELVEKLFAEDTEAKNPFSTQDTDLDLEMLAPYIPMDDDFQLRSFDQLSPLESSSASPESASPQSTVTVFQQTQIQEPTANATTTTATTDELKTVTKDRMEDIKILIASPSPTHIHKETTSATSSPYRDTQSRTASPNRAGKGVIEQTEKSHPRSPNVLSVALSQR | (5) |
| Securin | 202 | 39.7 | MATLIYVDKENGEPGTRVVAKDGLKLGSGPSIKALDGRSQVSTPRFGKTFDAPPALPKATRKALGTVNRATEKSVKTKGPLKQKQPSFSAKKMTEKTVKAKSSVPASDDAYPEIEKFFPFNPLDFESFDLPEEHQIAHLPLSGVPLMILDEERELEKLFQLGPPSPVKMPSPPWESNLLQSPSSILSTLDVELPPVCCDIDI | (17) |
| sml1 | 104 | 23.4 | MQNSQDYFYAQNRCQQQQAPSTLRTVTMAEFRRVPLPPMAEVPMLSTQNSMGSSASASASSLEMWEKDLEERLNSIDHDMNNNKFGSGELKSMFNQGKVEEMDF | (18) |
| PGR | 135 | 37.7 | AEPGKPAEPGKPAEPGKPAEPGTPAEPGKPAEPGTPAEPGKPAEPGKPAEPGKPAEPGKPAEPGTPAEPGTPAEPGKPAEPGTPAEPGKPAEPGTPAEPGKPAESGKPVEPGTPAQSGAPEQPNRSMHSTDNKNQ | (4) |
| Aβ(1-40) | 40 | 14.36 | DAEFRHDSGYEVHHQKLVFFAEDVGSNKGAIIGLMVGGVV | (19) |

**Table S2. List of proteins that exhibit phase separation behavior.**

| **Name** | **Database *^a^*** | **UniProt accession number** | **ID regions (*N*) *^b^*** | **folded regions (*N*) *^c^*** | **PDB entries** |
| --- | --- | --- | --- | --- | --- |
| TAF15 | Vernon *et al* | Q92804 | 1-36 (36)  62-207 (146)  210-234 (25)  379-398 (20) | 231-323 (93) | 2mmy.pdb |
| ROA1 | Vernon *et al* | P09651-2 | 183-216 (34)  297-320 (24) | 8-181 (174) | 1l3k.pdb |
| laf1 | Vernon *et al* | D0PV95 | 1-194 (194)  629-708 (80) | none | N/A |
| FUS | Vernon *et al* | H3BNZ4 | 1-263 (263) | none | N/A |
| DDX3X | Vernon *et al* | O00571 | 20-133 (114)  581-637 (57) | 134-580 (447) | 5e7i.pdb  2i4i.pdb |
| DDX4 | Vernon *et al* | Q9NQI0 | 26-250 (225)  694-724 (31) | none | N/A |
| eIF4H | Vernon *et al* | Q15056 | 10-39 (30)  117-152 (36)  159-248 (90) | none | N/A |
| NSP1 | Vernon *et al* | P14907 | 1-629 (629) | none | N/A |
| EWS | Vernon *et al* | F8WC90 | 126-164 (39)  177-292 (116) | none | N/A |
| TIA1 | Vernon *et al* | P31483 | 338-386 (49) | 2-39 (38)  44-85 (42)  105-285 (181) | 6eld.pdb  2mjn.pdb |
| Elastin | Vernon *et al* | P15502 | 466-488 (23)  611-651 (41) | none | N/A |
| ROA2 | Vernon *et al* | P22626-2 | 184-221 (38) | 3-183 (181) | 5en1.pdb |
| fib1 | Vernon *et al* | P22232 | 51-77 (27) | none | N/A |
| pgl-3 | Vernon *et al* | G5EBV6 | 445-466 (22)  519-589 (71)  616-693 (78) | none | N/A |
| CIRBP | Vernon *et al* | Q14011 | 86-172 (87) | 3-85 (83) | 5tbx.pdb |
| thermoNup98 | Vernon *et al* | D3KYQ3 | 88-120 (33)  125-167 (43) | none | N/A |
| tbNup158 | Vernon *et al* | Q387F2 | 1-50 (50)  120-147 (28) | none | N/A |
| Silk (spidroin-1) | Vernon *et al* | Q64K55 | 77-96 (20) *^e^* | 122-259 (138) *^f^* | 2mu3.pdb |
| Nup100 | Vernon *et al* | Q02629 | 20-49 (30)  215-238 (24)  357-384 (28)  416-438 (23)  445-465 (21)  763-789 (27) | none | N/A |
| Nup116 | Vernon *et al* | Q02630 | 259-279 (21)  475-527 (53)  904-942 (39) | 967-1111 (145) | 3pbp.pdb |
| Nup98B | Vernon *et al* | F4ID16 | 373-406 (34)  421-459 (39)  767-836 (70) | none | N/A |
| ddNup220 | Vernon *et al* | Q54EQ8 | 89-130 (42)  284-341 (58)  358-432 (75)  437-520 (84)  534-559 (26)  887-925 (39)  928-1017 (90)  1175-1198 (24) | none | N/A |
| TDP43 | Vernon *et al* | Q13148 | 344-371 (28) | 2-79 (78)  103-180 (78)  320-343 (24) | 5mdi.pdb  4y0f.pdb  2n3x.pdb |
| xNup214 | Vernon *et al* | Q9PVZ2 | 987-1032 (46)  1193-1213 (21)  1479-1503 (25) | none | N/A |
| ceNup98 | Vernon *et al* | G5EEH9 | 715-737 (24) | none | N/A |
| Nup98 | Vernon *et al* | P52948 | 521-565 (45)  886-940 (55) | 158-213 (56)  729-887 (159) | 3mmy.pdb  1ko6.pdb  2q5x.pdb |
| Nup153 | Vernon *et al* | P49790 | 98-123 (27)  404-424 (21)  1316-1340 (25)  1360-1386 (27) | none | N/A |
| xNup58 | Vernon *et al* | Q5EAX5 | none | 283-406 (124) | 5c3l.pdb |
| m. BugZ | Vernon *et al* | Q9JMD0-3 | 91-185 (95)  206-464 (259) | none | N/A |
| bfNup98 | Vernon *et al* | C3XWA2 | 636-677 (42)  691-716 (26)  876-979 (104)  1016-1039 (24) | none | N/A |
| xNup98 | Vernon *et al* | J7I6Y1 | 618-667 (50)  877-926 (93)  938-960 (23) | 716-866 (151) | 5e0q.pdb |
| dmNup98 | Vernon *et al* | Q9VCH5 | 703-802 (100) | none | N/A |
| xNup153 | Vernon *et al* | K9ZRR1 | 394-418 (25) | none | N/A |
| RBM14 | Vernon *et al* | Q96PK6 | none | 77-153 (77) | 2dnp.pdb |
| xNup54 | Vernon *et al* | K9ZTJ6 | none | 214-450 (237) | 5c2u.pdb  5c3l.pdb |
| x. BugZ | Vernon *et al* | Q7ZXV8 | 91-156 (66)  185-224 (40)  277-323 (47)  326-409 (84)  413-445 (33) | none | N/A |
| xNup62 | Vernon *et al* | Q91349 | none | 358-485 (128) | 5c3l.pdb |
| xPom121 | Vernon *et al* | Q5EWX9 | 439-466 (28) | none | N/A |
| WHI3 | Vernon *et al* | P34761 | 244-280 (37)  379-413 (35)  446-503 (62)  524-543 (20) | none | N/A |
| x1CG1 | Vernon *et al* | Q5XGN1 | none | none | N/A |
| xNup50 | Vernon *et al* | Q6DEC7 | 287-320 (34) | none | N/A |
| meg-3 | Vernon *et al* | Q9TXM1 | 1-38 (38)  84-105 (22)  150-177 (28)  271-295 (25)  473-493 (21)  508-536 (29) | none | N/A |
| eIF4G2 | Vernon *et al* | P39936 | 1-85 (85)  127-154 (28)  163-225 (63)  240-298 (59)  308-339 (32)  479-522 (44)  823-914 (92) | none | N/A |
| FMRP | PhaSePro | Q06787 | 434-632 (199) | 2-207 (206)  219-425 (207) | 4qvz.pdb  2qnd.pdb |
| TDP43 | PhaSePro | Q13148 | 270-306 (37)  350-374 (25)  384-414 (31) | 2-79 (78)  102-269 (168)  307-349 (43) | 5mdi.pdb  4bs2.pdb  2n2c.pdb |
| Nephrin | PhaSePro | O60500 | 478-502 (25)  1024-1061 (38)  1095-1229 (135) | none | N/A |
| N-WASP | PhaSePro | O00401 | 140-163 (24)  174-206 (33)  271-505 (235) | 207-270 (64) | 2lnh.pdb |
| NCK1 | PhaSePro | P16333 | 252-271 (20) | 4-59 (56)  101-163 (63)  281-377 (97) | 5qu2.pdb  2cub.pdb  2ci8.pdb |
| NUP98 | PhaSePro | P52948 | 39-81 (43)  103-157 (55)  214-241 (28)  254-346 (93)  375-434 (60)  447-591 (145)  607-686 (80)  698-728 (31)  883-982 (100)  995-1038 (44)  1069-1120 (51) | 158-213 (56)  729-880 (152) | 3mmy.pdb  2q5y.pdb |
| HP1α | PhaSePro | P45973 | 72-110 (39) | 17-68 (52)  111-170 (60) | 3fdt.pdb  3i3c.pdb |
| NPM1 | PhaSePro | P06748 | 120-239 (120) | 14-119 (106)  240-294 (55) | 5ehd.pdb  2llh.pdb |
| UBQLN2 | PhaSePro | Q9UHD9 | 104-143 (40)  167-189 (23)  200-353 (154)  369-473 (104)  483-624 (142) | 1-103 (103) | 1j8c.pdb |
| HSPB2 | PhaSePro | Q16082 | 1-27 (27)  162-182 (21) | 70-149 (80) | 6f2r.pdb, chain c |
| MAPT | PhaSePro | P10636-8 | 1-343 (343)  356-430 (75) | none | N/A |
| TNRC6B | PhaSePro | Q9UPQ9 | 1-1269 (1269)  1295-1638 (344)  1724-1764 (41)  1775-1833 (59) | none | N/A |
| Galectin-3 | PhaSePro | P17931 | 1-105 (105) | 113-250 (138) | 1kjl.pdb |
| p62 | PhaSePro | Q13501 | 194-388 (195) | 4-26 (23)  41-89 (49)  126-169 (44)  389-436 (48) | 6jm4.pdb  6jm4.pdb  5yp7.pd  1q02.pdb |
| LAT | PhaSePro | O43561 | 61-121 (61)  136-262 (127) | none | N/A |
| GRB2 | PhaSePro | P62993 | none | 1-217 (217) | 1gri.pdb |
| SOS1 | PhaSePro | Q07889 | 1047-1333 (287) | 6-1046 (1041) | 3ksy.pdb  1nvv.pdb |
| NONO | PhaSePro | Q15233 | 1-65 (65)  305-471 (167) | 66-304 (239) | 3sde.pdb |
| SFPQ | PhaSePro | P23246 | 1-278 (278)  598-707 (110) | 279-597 (319) | 6ncq.pdb  4wik.pdb |
| SYN1 | PhaSePro | P17600 | 1-115 (115)  380-705 (326) | none | N/A |
| cGAS | PhaSePro | Q8N884 | 1-153 (153) | 154-522 (369) | 4lev.pdb |
| MED1 | PhaSePro | Q15648 | 535-1581 (1047) | none | N/A |
| BRD4 | PhaSePro | O60885 | 1-43 (43)  173-346 (174)  464-600 (137)  684-1351 (668) | 44-172 (129)  347-463 (117)  601-683 (83) | 5u2e.pdb  6ffd.pdb  6bnh.pdb |
| Sam68 | PhaSePro | Q07666 | 1-98 (98)  183-219 (37)  274-440 (167) | 99-135 (37) | 2xa6.pdb |
| HNRNPD | PhaSePro | Q14103 | 1-95 (95)  267-290 (24) | 98-175 (78)  181-259 (79) | 1hd0.pdb  5im0.pdb  1wtb.pdb |
| SPOP | PhaSePro | O43791 | none | 28-356 (329) | 3hqi.pdb  4j8z.pdb |
| DAXX | PhaSePro | Q9UER7 | 5-54 (50)  143-182 (40)  387-740 (354) | 55-140 (86)  183-386 (204) | 5y18.pdb  4h9n.pdb |
| RPB1 | PhaSePro | P24928 | 36-60 (25)  322-355 (34)  605-624 (20)  718-759 (42)  1500-1970 (471) | none | N/A |
| Cyclin-T1 | PhaSePro | O60563 | 266-286 (21)  297-726 (430) | 4-263 (260) | 3blh.pdb  2pk2.pdb |
| DYRK1A | PhaSePro | Q13627 | 27-89 (63)  114-133 (20)  482-762 (281) | 134-481 (348) | 2vx3.pdb |
| PrP | PhaSePro | P04156 | 22-115 (94) | 119-230 (112) | 1i4m.pdb  1fkc.pdb |
| CBX2 | PhaSePro | Q14781 | 63-275 (213)  286-516 (231) | 9-62 (54) | 5epk.pdb |
| TIS11B | PhaSePro | Q07352 | 44-112 (69)  217-251 (35)  263-329 (67) | none | N/A |
| GATA3 | PhaSePro | P23771 | 2-223 (222)  235-260 (26)  366-443 (78) | 261-365 (105) | 4hc9.pdb |
| ERα | PhaSePro | P03372 | 104-174 (71)  256-281 (26)  551-572 (22) | 180-252 (73)  305-548 (244) | 1hcp.pdb  2ocf.pdb |
| DYRK3 | PhaSePro | O43781 | 1-35 (35)  45-137 (93) | 138-532 (395) | 5y86.pdb |
| SYN2 | PhaSePro | Q92777 | 1-115 (115)  397-573 (177) | none | N/A |
| PML | PhaSePro | P29590-12 | 1-30 (30)  434-563 (130) | 49-104 (56)  119-167 (49) | 1bor.pdb  6imq.pdb |
| PSD-95 | PhaSePro | P78352-3 | 35-54 (20) | 55-243 (189)  302-399 (98) | 6spv.pdb  3i4w.pdb |
| AGO2 | PhaSePro | Q9UKV8 | none | 22-859 (838) | 4f3t.pdb |
| Matrin-3 | PhaSePro | P43243 | 38-111 (74)  125-232 (108)  245-282 (38)  331-400 (70)  588-790 (203) | none | N/A |
| U2AF65 | PhaSePro | P26368 | 1-89 (89)  113-145 (33) | 90-112 (23)  148-336 (189)  375-475 (101) | 1jmt.pdb  2g4b.pdb  4fxw.pdb |
| MORC3 | PhaSePro | Q14149 | 455-660 (206)  755-776 (22)  857-885 (29) | 9-454 (446) | 6o1e.pdb |
| YTHDF2 | PhaSePro | Q9Y5A9 | 1-48 (48)  217-394 (178) | 398-548 (151) | 4wqn.pdb |
| YTHDF1 | PhaSePro | Q9BYJ9 | 1-53 (53)  70-89 (20)  132-188 (57)  216-363 (148) | 364-558 (195) | 4rci.pdb |
| YTHDF3 | PhaSePro | Q7Z739 | 1-54 (54)  81-113 (33)  136-378 (243)  517-538 (22) | none | N/A |
| CPEB3 | PhaSePro | Q8NE35 | 1-372 (372)  407-433 (27) | 440-540 (101) | 2rug.pdb |
| 53BP1 | PhaSePro | Q12888 | 1-1247 (1247)  1260-1481 (222)  1621-1713 (93) | 1484-1603 (120)  1714-1972 (259) | 2g3r.pdb  1kzy.pdb |
| Amyloid-beta | PhaSePro | P05067 | 194-286 (93)  349-370 (22)  625-671 (47) | 28-123 (96)  287-342 (56)  371-566 (196)  672-699 (28) | 1mwp.pdb  1app.pdb  3nyl.pdb  1amb.pdb |
| UBQLN2 | DisProt | Q9UHD9 | 450-624 (175) | none | N/A |
| PAB1 | DisProt | P04147 | 419-503 (85) | none | N/A |
| mid1 | DisProt | P78953 | 1-452 (452) | none | N/A |
| RXRG | DisProt | P48443 | 2-127 (126) | none | N/A |
| EMB506 | DisProt | Q9SQK3 | 40-112 (73) | none | N/A |
| AKRP | DisProt | Q05753 | 37-231 (195) | none | N/A |

*^a^* List of phase separating proteins obtained from Vernon *et al* (20), the PhaSePro database (21), and IDPs annotated “liquid-liquid phase separation” (IDPO:00041) in the DisProt database (22). Duplicate entries were removed from the combined list. The proteins TAF15, FUS, EWS, DDX3X, DDX4, TIA1, Elastin, RBM14, ROA1, and ROA2, listed in Vernon *et al*, were removed from the PhaSePro human protein set. ROA1 and ROA2 were identified as HNRNPA1 and HNRNPA2B1, respectively, in PhaSePro. IDPs annotated “liquid-liquid phase separation” in DisProt and originating from FUS, laf1, ROA1, ROA2, and DDX4, listed in Vernon *et al*, and DAXX, p62, TDP43, Galectin-3, NPM1, and NCK1, listed in PhaSePro, were also removed from the combined set.

*^b^* ID regions (*N*≥20) were identified from sequence using the GeneSilico MetaDisorder Service that generates a consensus prediction based on 13 primary methods (23). When this service was not available, the IUPred2 long predictor (24) was used instead. PhaSePro already annotates proteins in its database for the presence of predicted IDRs, by using IUPred2, which we kept for our use here. In this list, the ID regions exclude residues that were verified as folded (see column 5; i.e., a position could not be classified as both ID and folded). IDRs obtained from DisProt were assumed to be fully ID, because DisProt is manually curated for verified cases of ID.

*^c^* Residue positions with resolved atomic coordinates in a PDB structure (x-ray or NMR) were used to verify regions (*N*≥20) that fold. Unresolved residue positions in a PDB structure were not classified as folded. Folded regions from different structures that overlapped were merged.

*^e^* The silk (spidroin-1) sequence at positions 77-96 is repeated at positions 477-496, 677-696, 877-896, 1277-1296, 1477-1496, 1677-1696, 1877-1896, 2077-2096, 2277-2296, 2477-2496 and 2677-2696. Only one copy of this sequence was kept in the testing set.

*^f^* The silk (spidroin-1) sequence at positions 122-259 is repeated at positions 322-459, 522-659, 722-859, 922-1059, 1122-1259, 1322-1459, 1522-1659, 1722-1859, 1922-2059, 2122-2259, 2322-2459, 2522-2659. Only one copy of this sequence was kept in the folded set.

**Table S3. Summary of Mann-Whitney U tests that compare mean *v_model_* (top) and mean β-turn propensity (bottom) in the null, testing, and folded sets.**

*v_model_*: U test w/ null *^a^* U test w/ folded *^a^*

null set - 2.5e-08

testing set 2.4e-04 7.9e-03

folded set 2.5e-08 -

β-turn propensity: U test w/ null *^a^* U test w/ folded *^a^*

null set - 4.5e-08

testing set 1.0e-06 <1.1e-16

folded set 4.5e-08 -

*^a^* one-tail p-value

**Table S4. Normalized frequency for β-turn.**

| **Amino Acid** | **Scale value *^a^*** |
| --- | --- |
| Alanine | 0.770 |
| Arginine | 0.880 |
| Asparagine | 1.280 |
| Aspartic Acid | 1.410 |
| Cysteine | 0.810 |
| Glutamine | 0.980 |
| Glutamic Acid | 0.990 |
| Glycine | 1.640 |
| Histidine | 0.680 |
| Isoleucine | 0.510 |
| Leucine | 0.580 |
| Lysine | 0.960 |
| Methionine | 0.410 |
| Phenylalanine | 0.590 |
| Proline | 1.910 |
| Serine | 1.320 |
| Threonine | 1.040 |
| Tryptophan | 0.760 |
| Tyrosine | 1.050 |
| Valine | 0.470 |

*^a^* From Levitt (25).

**Table S5. Structural properties of turn and non-turn ensembles.**

|  | **Non-Turn Ensemble** *^a^* | **β-Turn Ensemble** *^a^* |
| --- | --- | --- |
| Total ASA (Å^2^) | 738.3 ± 0.9 | 670.3 ± 0.5 |
| Hydrophobic ASA (Å^2^) | 536.4 ± 0.8 | 488.9 ± 0.5 |
| CHASA (Å^2^) | 353.6 ± 0.6 | 327.3 ± 0.6 |
| Hydrophobic ASA lost assuming backbone hydration (Hydrophobic ASA – CHASA, Å^2^) | 182.8 ± 1.0 | 161.6 ± 0.7 |
| Number of Backbone Hydration Waters (CHASA maximum is 55) | 44.4 ± 0.1 | 37.1 ± 0.1 |

*^a^* Uncertainties were calculated as the standard error of the mean.

**Table S6. List of proteins that exhibit phase separation behavior *in cellulo* that were found by *in vitro* characterization not to phase separate as purified proteins.**

| **Name *^a^*** | **UniProt accession number** |
| --- | --- |
| RBM3 | P98179 |
| ERF3 | P05453 |
| CPEB2 | Q7Z5Q1 |
| WASL | O08816 |
| FMR1 | Q06787 |
| LSM4 | P40070 |
| SynGap | J3QQ18 |
| SOS1 | Q07889 |
| PUB1 | P32588 |
| LAT | O43561-2 |
| Nephrin | O60500 |
| dcp2 | O13828 |
| pdc1 | O13892 |
| Disks4 | P78352 |
| GRB2 | P62993 |
| NCK1 | P16333 |
| edc3 | O94752 |
| npm1 | P07222 |

*^a^* List obtained from (20).

**Table S7. Summary of pair-wise Mann-Whitney U tests comparing the relative population (given by set percentage) of predicted PS region lengths, for lengths ranging from 1 to 150 residues.** The sequence sets that were compared are those described in Figure 5 in the main text. Here, we compare each set known to be enriched for LLPS (rows) to those sets that are not enriched for that property (columns). Reported values are one-tail p-values, where p-value < 0.05 indicates a statistically significant difference in the two distributions.

human proteome *^a^* DisProt *^b^* SCOPe *^c^*

in vitro LLPS sufficient < 1.1e-16 < 1.1e-16 < 1.1e-16

in vitro LLPS insufficient 4.2e-12 7.2e-13 < 1.1e-16

DisProt LLPS annotated < 1.1e-16 < 1.1e-16 < 1.1e-16

PhaSePro < 1.1e-16 < 1.1e-16 < 1.1e-16

*^a^* UniProt reference proteome UP000005640

*^b^* DisProt database minus LLPS annotated entries

*^c^* SCOPe database version 2.07

**Supporting Figures**


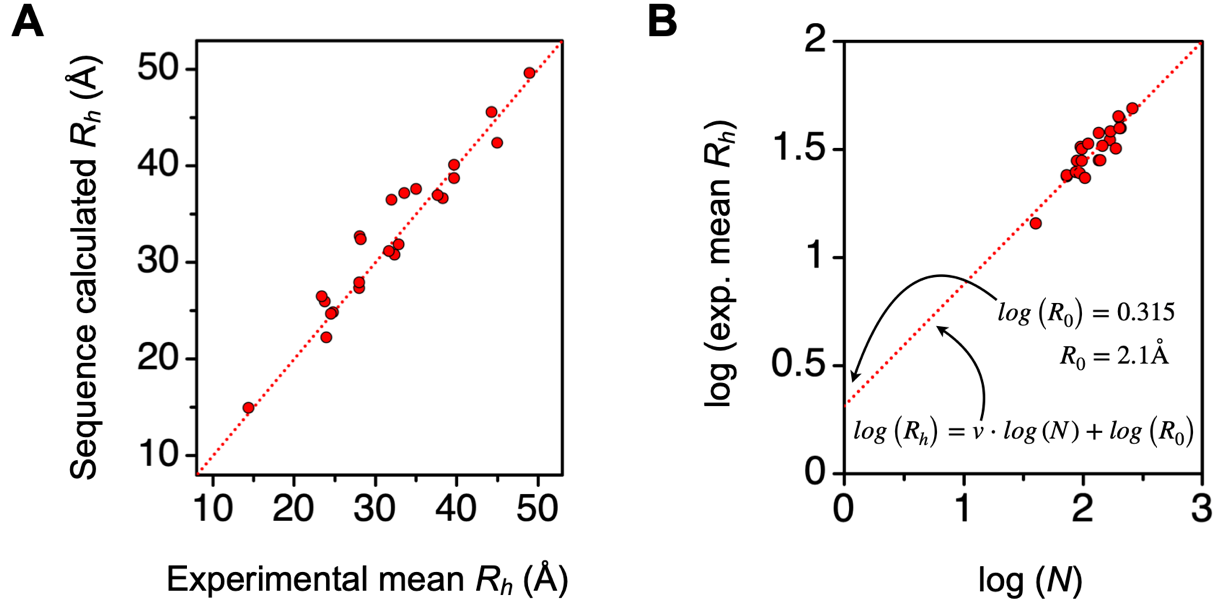


**Figure S1. Experimental mean *R_h_* compared to sequence calculated mean *R_h_*. A)** The identity of the IDPs, sequences, and their experimental values are provided in Table S1. Sequence calculated mean *R_h_* was determined using equation [3], given in Experimental Procedures. The stippled line is the identity line. **B)** The y-axis intercept from the trend line (stippled line in figure) of a log-log plot of mean *R_h_* and *N* (protein length) yields the pre-factor, *R_o_*, in the power law scaling equation *R_h_* = *R_o_*･*N^v^*.


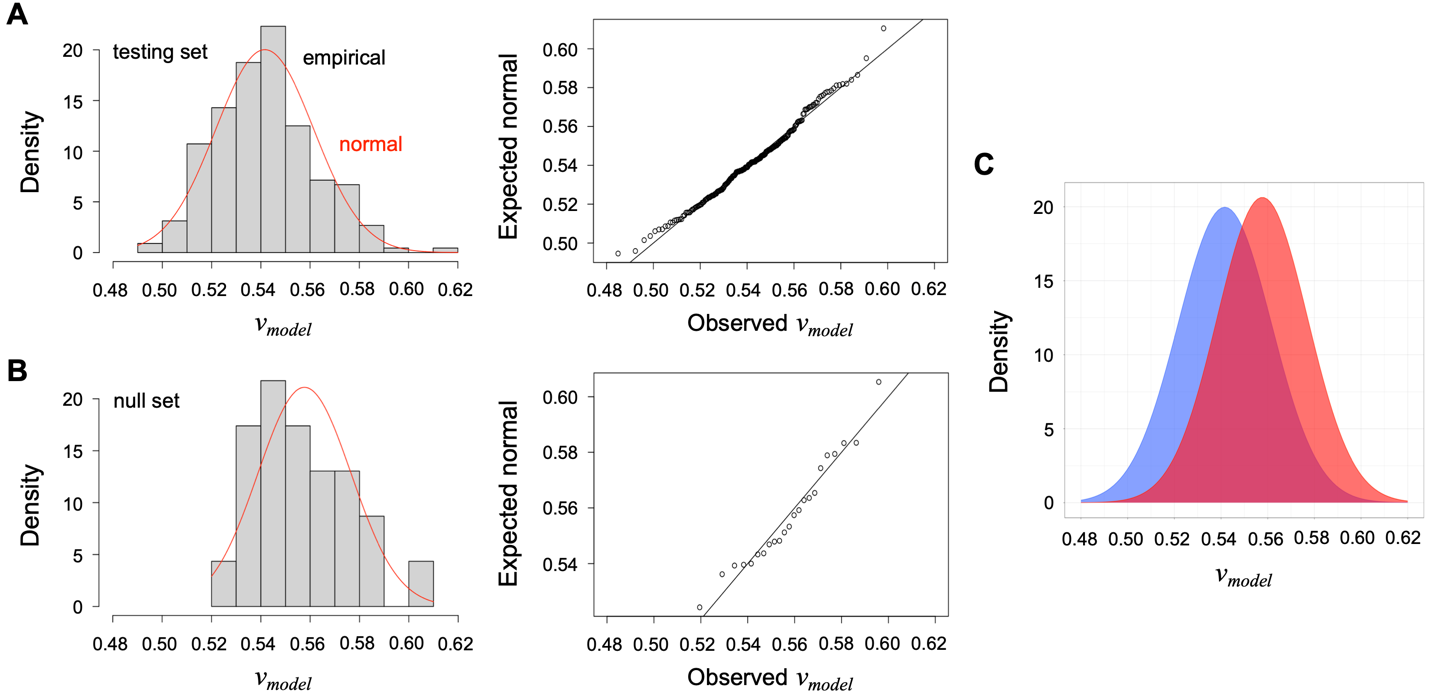


**Figure S2.** **Distribution of *v_model_* values in the A) testing and B) null sets.** The left-most figure in panels A and B compares the histogram distribution of *v_model_* values in the testing and null sets, respectively, to the probability density function of the normal distribution, $f\left( x \right)=\frac{1}{\sigma\sqrt{2\pi}}e^{-\frac{1}{2}\left( \frac{x-\mu}{\sigma} \right)^{2}}$, shown by the red line, where μ and σ are the distribution mean and standard deviation. The right-most figure is a Q-Q (quantile-quantile) plot that compares two probability distributions by plotting the quantiles against each other; in this case the observed empirical against the normal. When the trend in this plot follows the identity line (black line), this provides evidence that the compared distributions are similar. Because both the testing and null sets exhibit this behavior when compared to normal distributions, both data sets can be considered as similar to normal. Panel C overlays the distribution of *v_model_* values in the testing (blue) and null (red) sets when calculated as normal using the probability density function and their observed distribution mean and standard deviation.


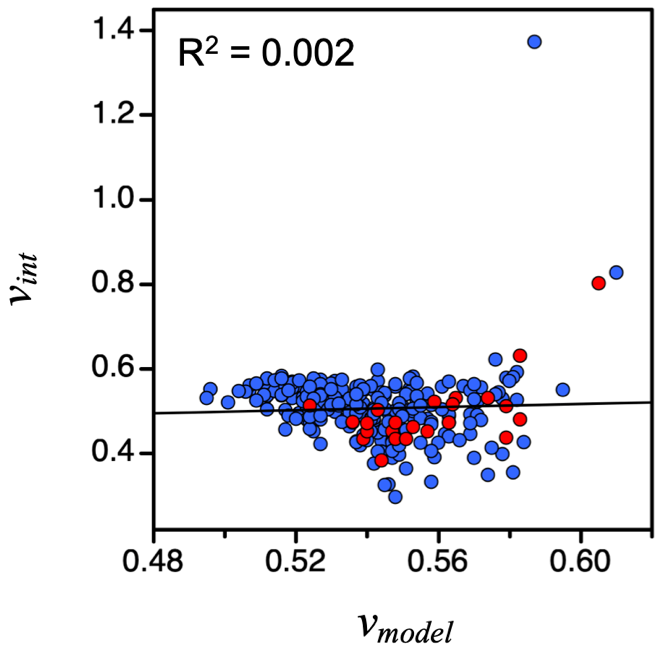


**Figure S3. Comparing sequence calculated *v_model_* and *v_int_*.** Blue circles show the calculated values of *v_model_* and *v_int_* for the IDR sequences in the testing set (**Table S2**). Red circles show values for the IDP sequences in the null set (**Table S1**). The correlation, R^2^, was calculated for the combined set of sequences, testing and null.


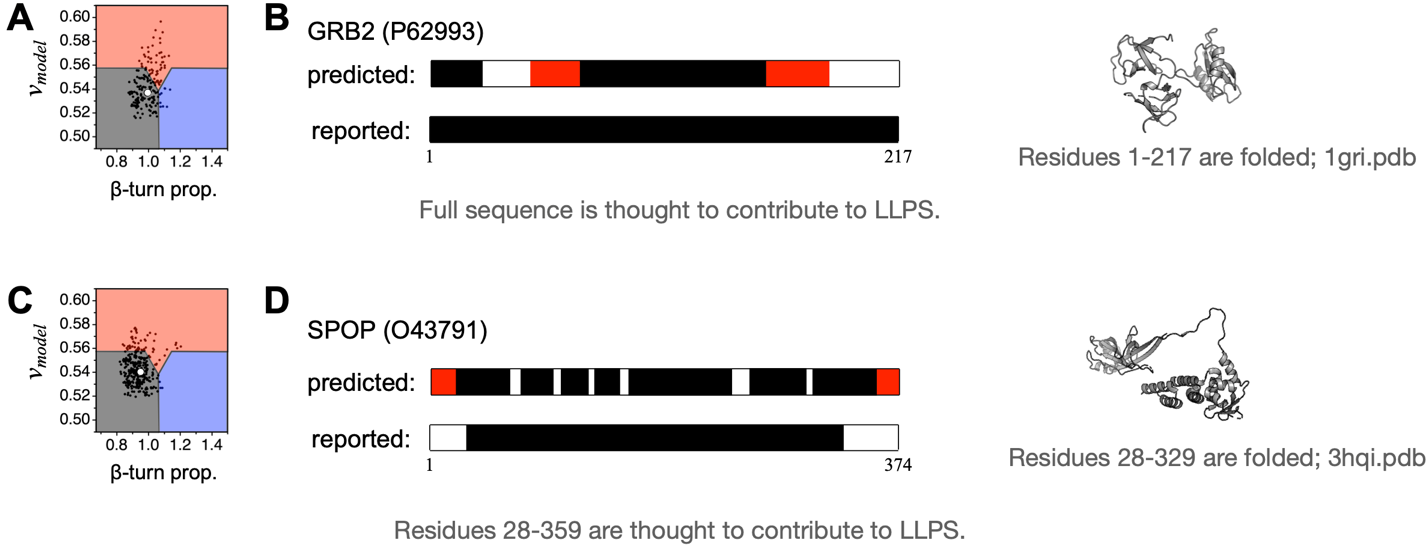


**Figure S4. Predicting protein regions that drive LLPS. A-D)** Using a sliding window algorithm applied to the primary sequence (described in Figure 4 legend of main text), contiguous regions (*N*≥20) that were 90% of only one label P, D, or F were colored blue, red, or black, respectively, to represent predicted PS, ID, or folded regions. The proteins in this figure are identified by name and UniProt accession number. The ParSe algorithm predicts there are no PS (i.e., phase separating ID) regions in these two proteins.


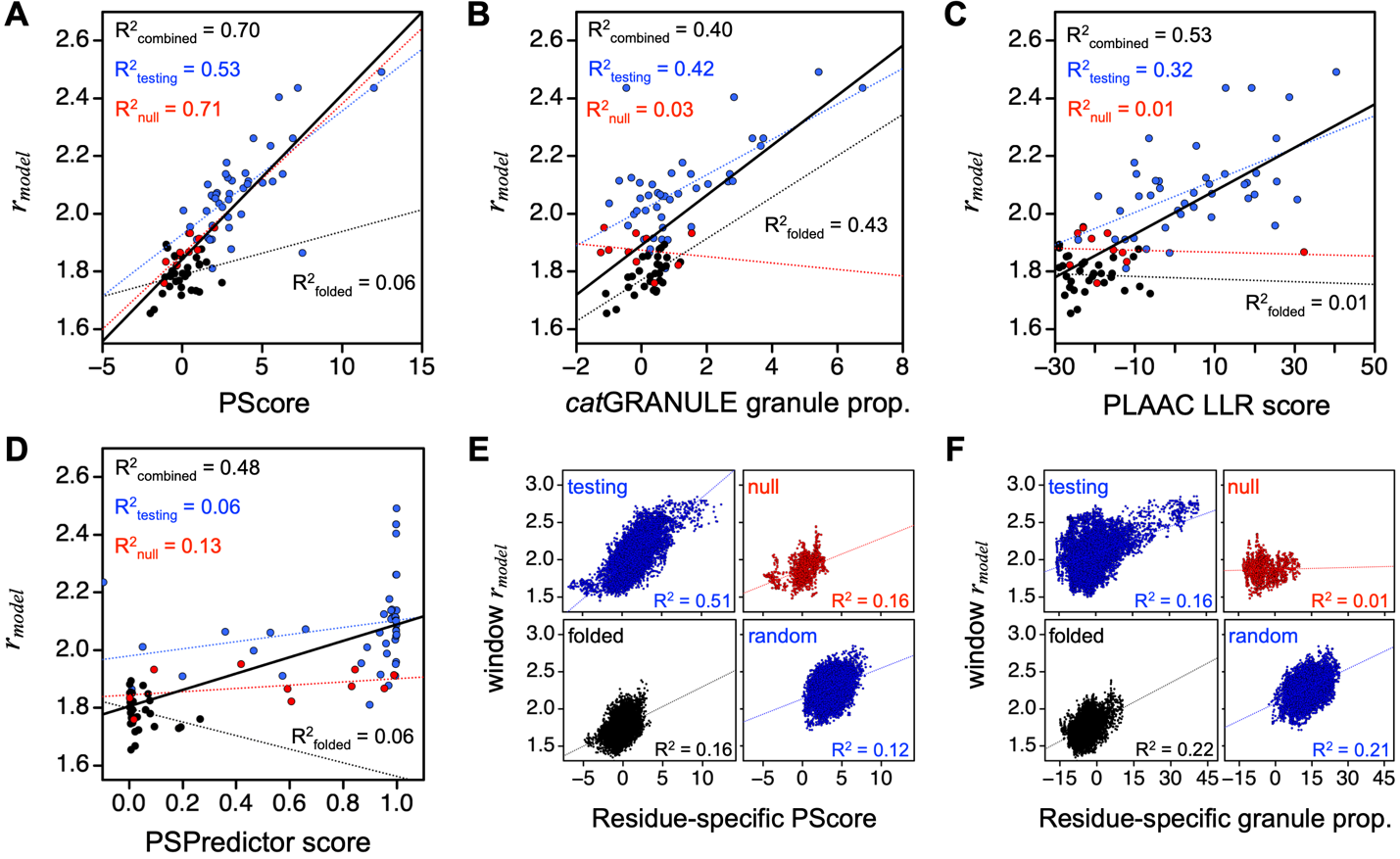


**Figure S5. Pair-wise correlations of predictor results.** Comparison of *r_model_* to **A)** PScore, **B)** granule propensity, **C)** LLR, and **D)** PSPredictor in the testing, null, folded, and combined sequence sets. Residue level comparison of window *r_model_*, where the window value was assigned to the central residue position, to **E)** PScore and **F)** granule propensity for sequences in the testing (top left), null (top right), folded (bottom left), and randomized (bottom right) sets.

**Supporting References**

1. Perez, R. B., Tischer, A., Auton, M., and Whitten, S. T. (2014) Alanine and proline content modulate global sensitivity to discrete perturbations in disordered proteins. *Proteins*. **82**, 3373–3384

2. Lowry, D. F., Stancik, A., Shrestha, R. M., and Daughdrill, G. W. (2008) Modeling the accessible conformations of the intrinsically unstructured transactivation domain of p53. *Proteins*. **71**, 587–598

3. Donaldson, L., and Capone, J. P. (1992) Purification and characterization of the carboxyl-terminal transactivation domain of Vmw65 from herpes simplex virus type 1. *J. Biol. Chem.* **267**, 1411–1414

4. English, L. R., Tilton, E. C., Ricard, B. J., and Whitten, S. T. (2017) Intrinsic α helix propensities compact hydrodynamic radii in intrinsically disordered proteins. *Proteins*. **85**, 296–311

5. Sánchez-Puig, N., Veprintsev, D. B., and Fersht, A. R. (2005) Binding of natively unfolded HIF-1alpha ODD domain to p53. *Mol. Cell*. **17**, 11–21

6. Campbell, K. M., Terrell, A. R., Laybourn, P. J., and Lumb, K. J. (2000) Intrinsic structural disorder of the C-terminal activation domain from the bZIP transcription factor Fos. *Biochemistry*. **39**, 2708–2713

7. Geething, N. C., and Spudich, J. A. (2007) Identification of a minimal myosin Va binding site within an intrinsically unstructured domain of melanophilin. *J. Biol. Chem.* **282**, 21518–21528

8. Soragni, A., Zambelli, B., Mukrasch, M. D., Biernat, J., Jeganathan, S., Griesinger, C., Ciurli, S., Mandelkow, E., and Zweckstetter, M. (2008) Structural characterization of binding of Cu(II) to tau protein. *Biochemistry*. **47**, 10841–10851

9. Adkins, J. N., and Lumb, K. J. (2002) Intrinsic structural disorder and sequence features of the cell cycle inhibitor p57Kip2. *Proteins*. **46**, 1–7

10. Uversky, V. N., Permyakov, S. E., Zagranichny, V. E., Rodionov, I. L., Fink, A. L., Cherskaya, A. M., Wasserman, L. A., and Permyakov, E. A. (2002) Effect of zinc and temperature on the conformation of the gamma subunit of retinal phosphodiesterase: a natively unfolded protein. *J. Proteome Res.* **1**, 149–159

11. Haaning, S., Radutoiu, S., Hoffmann, S. V., Dittmer, J., Giehm, L., Otzen, D. E., and Stougaard, J. (2008) An unusual intrinsically disordered protein from the model legume Lotus japonicus stabilizes proteins in vitro. *J. Biol. Chem.* **283**, 31142–31152

12. Permyakov, S. E., Millett, I. S., Doniach, S., Permyakov, E. A., and Uversky, V. N. (2003) Natively unfolded C-terminal domain of caldesmon remains substantially unstructured after the effective binding to calmodulin. *Proteins*. **53**, 855–862

13. Paleologou, K. E., Schmid, A. W., Rospigliosi, C. C., Kim, H.-Y., Lamberto, G. R., Fredenburg, R. A., Lansbury, P. T., Fernandez, C. O., Eliezer, D., Zweckstetter, M., and Lashuel, H. A. (2008) Phosphorylation at Ser-129 but not the phosphomimics S129E/D inhibits the fibrillation of alpha-synuclein. *J. Biol. Chem.* **283**, 16895–16905

14. Baker, J. M. R. (2009) *Structural Characterization and Interactions of the CFTR Regulatory Region*. Ph. D. thesis, Department of Biochemistry, University of Toronto, Toronto

15. Choi, U. B., McCann, J. J., Weninger, K. R., and Bowen, M. E. (2011) Beyond the random coil: stochastic conformational switching in intrinsically disordered proteins. *Structure*. **19**, 566–576

16. Magidovich, E., Orr, I., Fass, D., Abdu, U., and Yifrach, O. (2007) Intrinsic disorder in the C-terminal domain of the Shaker voltage-activated K+ channel modulates its interaction with scaffold proteins. *Proc. Natl. Acad. Sci. U.S.A.* **104**, 13022–13027

17. Sánchez-Puig, N., Veprintsev, D. B., and Fersht, A. R. (2005) Human full-length Securin is a natively unfolded protein. *Protein Sci.* **14**, 1410–1418

18. Danielsson, J., Liljedahl, L., Bárány-Wallje, E., Sønderby, P., Kristensen, L. H., Martinez-Yamout, M. A., Dyson, H. J., Wright, P. E., Poulsen, F. M., Mäler, L., Gräslund, A., and Kragelund, B. B. (2008) The intrinsically disordered RNR inhibitor Sml1 is a dynamic dimer. *Biochemistry*. **47**, 13428–13437

19. Danielsson, J., Jarvet, J., Damberg, P., and Gräslund, A. (2002) Translational diffusion measured by PFG-NMR on full length and fragments of the Alzheimer Aβ(1–40) peptide. Determination of hydrodynamic radii of random coil peptides of varying length. *Magnetic Resonance in Chemistry*. **40**, S89–S97

20. Vernon, R. M., Chong, P. A., Tsang, B., Kim, T. H., Bah, A., Farber, P., Lin, H., and Forman-Kay, J. D. (2018) Pi-Pi contacts are an overlooked protein feature relevant to phase separation. *Elife*. 10.7554/eLife.31486

21. Mészáros, B., Erdős, G., Szabó, B., Schád, É., Tantos, Á., Abukhairan, R., Horváth, T., Murvai, N., Kovács, O. P., Kovács, M., Tosatto, S. C. E., Tompa, P., Dosztányi, Z., and Pancsa, R. (2020) PhaSePro: the database of proteins driving liquid–liquid phase separation. *Nucleic Acids Res*. **48**, D360–D367

22. Hatos, A., Hajdu-Soltész, B., Monzon, A. M., Palopoli, N., Álvarez, L., Aykac-Fas, B., Bassot, C., Benítez, G. I., Bevilacqua, M., Chasapi, A., Chemes, L., Davey, N. E., Davidović, R., Dunker, A. K., Elofsson, A., Gobeill, J., Foutel, N. S. G., Sudha, G., Guharoy, M., Horvath, T., Iglesias, V., Kajava, A. V., Kovacs, O. P., Lamb, J., Lambrughi, M., Lazar, T., Leclercq, J. Y., Leonardi, E., Macedo-Ribeiro, S., Macossay-Castillo, M., Maiani, E., Manso, J. A., Marino-Buslje, C., Martínez-Pérez, E., Mészáros, B., Mičetić, I., Minervini, G., Murvai, N., Necci, M., Ouzounis, C. A., Pajkos, M., Paladin, L., Pancsa, R., Papaleo, E., Parisi, G., Pasche, E., Barbosa Pereira, P. J., Promponas, V. J., Pujols, J., Quaglia, F., Ruch, P., Salvatore, M., Schad, E., Szabo, B., Szaniszló, T., Tamana, S., Tantos, A., Veljkovic, N., Ventura, S., Vranken, W., Dosztányi, Z., Tompa, P., Tosatto, S. C. E., and Piovesan, D. (2020) DisProt: intrinsic protein disorder annotation in 2020. *Nucleic Acids Res.* **48**, D269–D276

23. Kozlowski, L. P., and Bujnicki, J. M. (2012) MetaDisorder: a meta-server for the prediction of intrinsic disorder in proteins. *BMC Bioinformatics*. **13**, 111

24. Erdős, G., and Dosztányi, Z. (2020) Analyzing Protein Disorder with IUPred2A. *Current Protocols in Bioinformatics*. **70**, e99

25. Levitt, M. (1978) Conformational preferences of amino acids in globular proteins. *Biochemistry*. **17**, 4277–4285
